# Phenotypic and immunological profile divergence between SLE-associated myelitis and SLE-NMOSD overlap syndrome: a retrospective case series

**DOI:** 10.3389/fimmu.2026.1925485

**Published:** 2026-10-20

**Authors:** Tingting Zhang, Qingmiao Liu, Hui Cong, Yiling Huang, Hui Zhang

**Affiliations:** Department of Neurology, First Affiliated Hospital of Dalian Medical University, Dalian, China

**Keywords:** aquaporin-4 antibody, inebilizumab, myelitis, neuromyelitis optica spectrum disorder, systemic lupus erythematosus

## Abstract

**Background:**

Systemic lupus erythematosus-associated myelitis (SLE-AM) is a devastating neuroimmunological complication. Increasing evidence suggests that myelitis occurring in patients with SLE is heterogeneous and may include non-overlap SLE-AM and SLE-neuromyelitis optica spectrum disorder (NMOSD) overlap syndrome. We aimed to describe the clinical, radiological, CSF inflammatory and systemic immunological profiles of patients with these conditions and to explore their potential therapeutic implications.

**Methods:**

We conducted a retrospective, single-center case series of six patients with SLE-associated myelitis treated at the First Affiliated Hospital of Dalian Medical University. Two patients were classified as having non-overlap SLE-AM and four as having SLE-NMOSD overlap syndrome according to clinical, radiological, and serological findings.

**Results:**

Among the six patients, five were women and one was a man; Patients 1 and 2 had non-overlap SLE-AM, whereas Patients 3–6 had SLE-NMOSD overlap syndrome. In Patients 1 and 2, myelitis occurred concurrently with SLE onset and was accompanied by marked cerebrospinal fluid (CSF) inflammation, high anti-dsDNA antibody levels, and complement consumption. Patients 3–6 had more heterogeneous temporal courses and generally lower routine CSF inflammatory indices; serum AQP4-IgG was positive in Patients 4–6 and was not tested in Patient 3. Despite intensive immunosuppressive treatment, several patients had substantial residual neurological disability during follow-up. Notably, Patient 4 showed improvement in left-leg pain and remained clinically stable, with no recurrence over nearly 7 months of follow-up after inebilizumab treatment.

**Conclusion:**

Non-overlap SLE-AM and SLE-NMOSD overlap syndrome may show differing clinical and CSF profiles. Detailed serological and CSF characterization may help distinguish these clinical patterns and inform more individualized therapeutic strategies.

## 1 Introduction

Systemic lupus erythematosus (SLE) is a multisystem autoimmune disease characterized by highly heterogeneous neuropsychiatric manifestations (1). SLE-associated myelitis (SLE-AM) is a rare complication affecting approximately 0.5% to 1.0% of patients and is the presenting feature in 30%-60% of cases (2). Yet it remains clinically devastating, frequently manifesting with profound motor paralysis, ascending sensory loss, and acute sphincter dysfunction (2). Historically, this condition was often attributed to vascular-thrombotic mechanisms driven by nonspecific vasculitis or antiphospholipid antibodies (2, 3). However, available evidence suggests that antiphospholipid antibody status does not consistently predict neurological outcome, and empiric anticoagulation has not shown clear benefit (3). These findings imply that non-thrombotic mechanisms actively contribute to the pathogenesis of spinal cord damage in SLE. Consistent with this, Birnbaum et al. (4) demonstrated that SLE-AM encompasses two highly divergent clinical phenotypes regarding magnetic resonance imaging (MRI) features, further arguing against a single vascular etiology and instead pointing toward distinct immunopathological mechanisms. Pooled cohort-level data and other observations further support that demyelinating syndromes in SLE are heterogeneous rather than monomorphic (5).

The discovery of aquaporin-4 immunoglobulin G (AQP4-IgG) as a serum biomarker for neuromyelitis optica spectrum disorder (NMOSD) fundamentally refined the interpretation of spinal cord syndromes previously grouped under lupus myelitis (6, 7). AQP4-IgG serves as an important diagnostic biomarker, as it is robustly detectable in rheumatologic patients presenting with NMOSD but is virtually absent in patients with other neuropsychiatric SLE (NPSLE) presentations (8). More recent biomarker-era reassessment of rheumatologic myelitis has reinforced that a substantial proportion of cases historically labeled ‘lupus myelitis’ are better understood as AQP4-IgG-positive NMOSD or related inflammatory demyelinating syndromes (9). Furthermore, recent Mendelian randomization evidence suggests that SLE susceptibility increases the risk of developing NMOSD and that their clinical intersection may reflect shared genetic and immunopathological architecture (10). Consequently, the classification of myelitis in SLE has evolved beyond purely anatomical descriptions to incorporate serological markers, highlighting that myelitis in patients with SLE is heterogeneous and may include non-overlap SLE-AM and NMOSD overlap.

Despite the need for precise clinical characterization, direct patient-level data describing the clinical course and inflammatory profiles of non-overlap SLE-AM and SLE-NMOSD overlap syndrome remain scarce. Because cerebrospinal fluid (CSF) can provide information about localized central nervous system inflammation, detailed assessment of the CSF profile may help characterize these presentations. In this retrospective case series, we described the clinical, radiological, immunological, treatment, and outcome characteristics of six patients with SLE-associated myelitis. We further examined patient-level clinical, CSF inflammatory, and systemic immunological profiles across these presentations to explore potentially differing patterns. We also examined longitudinal treatment courses to explore the potential implications of these patterns for individualized therapeutic selection.

## 2 Methods

### 2.1 Study design and patient selection

This retrospective, single-center case series was conducted at the First Affiliated Hospital of Dalian Medical University. We retrospectively searched the hospital electronic medical record system for all patients diagnosed with both SLE and myelitis between January 2013 and March 2026. A total of seven patients were identified. One patient was excluded from the final analysis due to incomplete clinical documentation that precluded the reconstruction of baseline characteristics. Six patients met the prespecified eligibility criteria and were included in the final case series. Eligible patients were required to have: (i) a definite diagnosis of SLE; (ii) inflammatory spinal cord dysfunction supported by neurological evaluation and spinal cord MRI; and (iii) sufficient clinical documentation to reconstruct baseline features, hospital-based treatments, and clinical outcomes. Patients whose myelitis was more likely explained by compressive, infectious, vascular, neoplastic, metabolic, or other non-inflammatory causes were excluded after chart review. Given the retrospective design and the use of anonymized clinical data, the requirement for informed consent was waived by the Institutional Ethics Committee (Approval No. PJ-KS-BD-2026-17), consistent with the principles of the Declaration of Helsinki.

### 2.2 Diagnostic framework

The diagnosis of SLE was retrospectively confirmed according to the 2019 EULAR/ACR classification criteria (11). For descriptive purposes, cases were classified as non-overlap SLE-AM or SLE-NMOSD overlap syndrome. Non-overlap SLE-AM was defined by the absence of clinical, radiological, or serological evidence supporting coexisting NMOSD during the documented observation period. SLE-NMOSD overlap syndrome was defined by fulfillment of diagnostic criteria for both SLE and NMOSD based on the available clinical, serological, and MRI findings. NMOSD was classified according to the 2015 International Panel for NMO Diagnosis criteria (IPND) (7). Serum MOG-IgG was negative in all patients who were tested, with the diagnostic assessment aligned with contemporary MOGAD criteria (12). Myelitis was defined pragmatically as inflammatory spinal cord dysfunction with motor, sensory, and/or sphincter disturbance supported by compatible MRI abnormalities and/or CSF inflammatory findings after exclusion of alternative etiologies. Furthermore, for all applicable patients in this case series, AQP4-IgG and MOG-IgG testing was performed using a fixed cell-based assay (CBA) on serum and CSF samples. Serum and CSF specimens were collected concurrently on the day of lumbar puncture. No patient had received intravenous immunoglobulin or plasma exchange before paired sample collection. AQP4-IgG and MOG-IgG testing was not performed in Patient 3 because antibody testing was not available at the time of her index neurological evaluation. Nevertheless, she fulfilled the 2015 IPND diagnostic criteria for NMOSD based on the combination of recurrent longitudinally extensive transverse myelitis (LETM), optic neuritis, characteristic MRI findings, and the exclusion of alternative demyelinating or vascular etiologies.

### 2.3 Data acquisition and variable definition

We extracted clinical, laboratory, MRI, and treatment data from the electronic medical record system. Demographic and temporal variables included sex, age at SLE and myelitis onset, and disease duration prior to the index event. For all six patients, the CSF sample analyzed in this study was obtained during the first documented episode of myelitis. Clinical assessment encompassed the time to neurological nadir, initial motor, sensory, and sphincter disturbances, and clinical status at the last documented encounter. Longitudinal neurological outcomes were extracted from the available medical records, including Medical Research Council (MRC) muscle strength, ambulatory status, sensory symptoms or signs, visual symptoms, sphincter function, documented relapses, and follow-up MRI or AQP4-IgG results. Furthermore, the timing of cerebrospinal fluid (CSF) collection by lumbar puncture (LP) relative to symptom onset and prior baseline immunosuppressive exposures were systematically documented and evaluated. Radiological assessment included lesion location and longitudinal extent, axial cord distribution, gray/white matter predominance, enhancement pattern, cord swelling, and associated brain and optic nerve abnormalities when available. LETM was defined as a lesion spanning at least three vertebral segments. Treatment courses and clinical outcomes at discharge and the last documented follow-up were summarized individually for each patient. Data were summarized descriptively at the individual-patient level.

## 3 Results

### 3.1 Clinical and demographic profiles

Six patients were included, comprising five women and one man. Patients 1 and 2 had non-overlap SLE-AM, whereas Patients 3–6 had SLE-NMOSD overlap syndrome (**Table 1**).

**Table 1:** Clinical, immunological, and radiological profiles of individual patients.

Characteristics	Non-overlap SLE-AM		SLE-NMOSD overlap			
	Patient 1	Patient 2	Patient 3	Patient 4	Patient 5	Patient 6
Clinical and immunological profile						
Gender	Female	Male	Female	Female	Female	Female
Age at myelitis onset, years	20	31	37	63	59	19
Age at SLE onset, years	20	31	35	63	44	14
Disease duration prior to myelitis, years	0	0	1.8	0	14.5	4.5
Time to nadir	1 day	4 months	1 month	1 month	1.7 months	5 months
Sphincter dysfunction	(+)	(+)	(-)	(-)	(-)	(-)
AQP4-IgG (Serum)	(-)	(-)	ND	1:32	1:32	1:32
MOG-IgG (Serum)	(-)	(-)	ND	(-)	(-)	(-)
AQP4-IgG (CSF)	(-)	(-)	ND	(-)	(-)	(-)
MOG-IgG (CSF)	(-)	(-)	ND	(-)	(-)	(-)
Radiological profiles						
LETM (≥3 segments)	Yes	Yes	Yes	Yes	Yes	No
Spinal segments involved	C1-C4, C7-T3, T7-T12, L1	C3-C6, T9-T10	C6-T4	T4-T6	T2-T9	C3-C4
Axial cord location	Central gray and surrounding white matter involvement	Central gray and surrounding white matter involvement	Central gray matter predominance	Central gray matter	Central gray matter	Central gray and surrounding white matter involvement
Gray/white matter predominance	Mixed	Mixed	Gray-matter predominant	H-sign (H-shaped gray matter involvement)	(bilateral horns)	Mixed
Spinal cord enhancement pattern	No abnormal enhancement	No abnormal enhancement	ND	No abnormal enhancement	No abnormal enhancement	No abnormal enhancement
Spinal cord swelling	Moderate to marked swelling	Mild swelling	Moderate swelling	Moderate swelling	Moderate swelling	Marked swelling
Brain abnormalities	Medullary hyperintensity	No abnormal signal intensity	No abnormal signal intensity	right parietal lobe hyperintensity	No abnormal signal intensity	Brainstem, hippocampus, right temporal, and frontal lobes with patchy or incomplete ring-like enhancement
Optic nerve abnormalities	ND	ND	ND	ND	ND	No structural abnormality (despite clinical visual blurring)

AQP4-IgG and MOG-IgG were tested in paired serum and CSF samples using a fixed cell-based assay (CBA). Serum and CSF specimens were collected concurrently on the day of LP.

AQP4-IgG, aquaporin-4 immunoglobulin G; LETM, longitudinally extensive transverse myelitis; MOG-IgG, myelin oligodendrocyte glycoprotein immunoglobulin G; NMOSD, neuromyelitis optica spectrum disorder; SLE-AM, systemic lupus erythematosus-associated myelitis; SLE-NMOSD overlap, systemic lupus erythematosus and neuromyelitis optica spectrum disorder overlap syndrome. Data notation: (+), positive; (-), negative. ND, not done.

In Patients 1 and 2, myelitis occurred concurrently with the initial presentation of SLE. Patient 1 reached the neurological nadir within 1 day, whereas Patient 2 reached the nadir over 4 months; both patients had sphincter dysfunction. Patient 2 developed progressive lower-limb weakness followed by bowel and sensory symptoms and had received no disease-specific immunotherapy before evaluation. Despite the prolonged course, inflammatory myelitis was supported by progressive neurological deficits, multisegmental spinal cord lesions, and inflammatory CSF findings (WBC 23 ×10^6^/L; protein 1.774 g/L). Among Patients 3–6, the interval from SLE onset to myelitis and the time to neurological nadir varied considerably (**Table 1**). None had documented sphincter dysfunction at the initial neurological presentation.

### 3.2 Radiological profiles

MRI findings across the six patients were heterogeneous and are systematically summarized in **Table 1**, including longitudinal lesion extent, axial distribution, gray/white matter predominance, enhancement pattern, cord swelling, and associated brain or optic nerve abnormalities. Sagittal and corresponding axial spinal MRI images from all six patients are shown in **Figure 1**. Patients 1 and 2 both had extensive spinal cord involvement and met the criteria for LETM. Patient 1 had lesions involving C1–C4, C7–T3, T7–T12, and L1, whereas Patient 2 had lesions involving C3–C6 and T9–T10. The extensive, multisegmental spinal cord involvement in Patients 1 and 2 coincided with sphincter dysfunction in both patients. Among Patients 3–6, Patients 3, 4, and 5 also had LETM, involving C6–T4, T4–T6, and T2–T9, respectively, whereas Patient 6 had a shorter lesion involving C3–C4. Overall, the lesion distribution and extent among Patients 3–6 were more heterogeneous than those observed in Patients 1 and 2.

**Figure 1:**
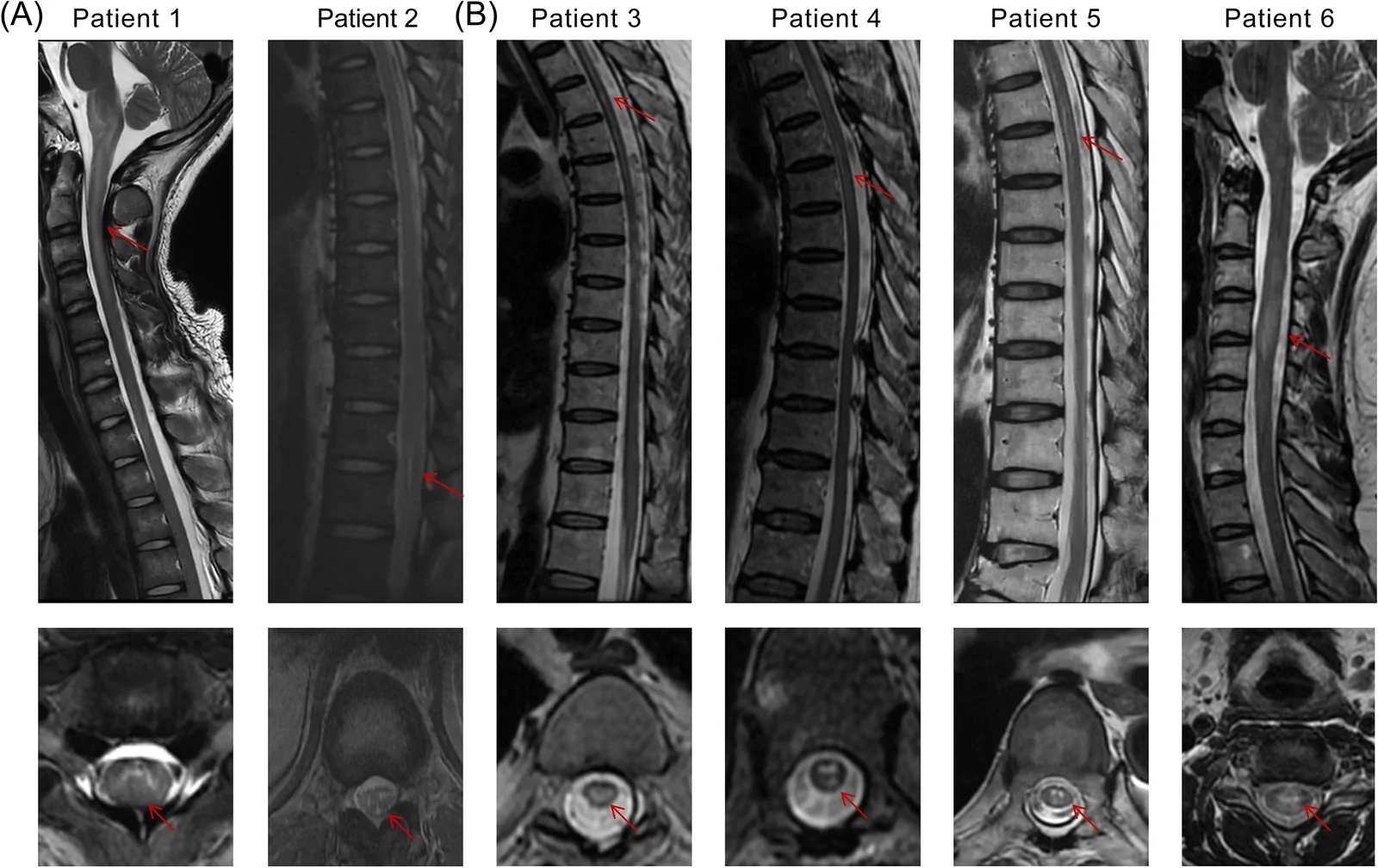
Spinal cord MRI findings in all six patients with SLE-associated myelitis. **(A)** Patients 1 and 2 with non-overlap SLE-associated myelitis (SLE-AM). **(B)** Patients 3–6 with SLE–neuromyelitis optica spectrum disorder (NMOSD) overlap syndrome. For each patient, sagittal spinal cord MRI images are shown in the upper row and corresponding axial images in the lower row. Red arrows indicate intramedullary spinal cord abnormalities. The complete documented distribution of spinal cord involvement for each patient is summarized in **Table 1**.

### 3.3 Immunological and inflammatory patterns

The clinical and radiological heterogeneity among the six patients was accompanied by differences in CSF and systemic serological profiles (**Table 2**). At the available sampling time points, Patients 1 and 2 had higher CSF leukocyte counts and protein concentrations than Patients 3–6. The interval from neurological symptom onset to LP varied substantially among patients, ranging from 5 days to 4.3 months, and pre-LP immunotherapy exposure also differed across individuals. CSF glucose and chloride levels were broadly comparable, whereas opening pressure varied without a consistent pattern across patients.

**Table 2:** Serological and CSF inflammatory profiles of individual patients.

Characteristics	Non-overlap SLE-AM		SLE-NMOSD overlap			
	Patient 1	Patient 2	Patient 3	Patient 4	Patient 5	Patient 6
CSF inflammatory profiles						
Pressure, mmH_2_O	250	210	150	150	140	200
Protein, g/L	0.830	1.774	0.573	0.600	0.607	0.300
WBC, ×10^6^/L	73	23	14	6	9	6
Total cell count, ×10^6^/L	318	28	17	22	9	10
Glucose, mmol/L	2.61	2.04	2.07	2.71	3.48	4.0
Chloride, mmol/L	122	126	124	126	124	122
Time from symptom onset to LP	5 days	4.3 months	1.3 months	1.1 months	1.8 months	0.5 months
Immunotherapy before LP	High-dose pulse (500mg)	Untreated	Oral prednisone and CTX	None	Oral prednisone	Oral prednisone + MMF
Systemic serological profiles						
ESR, mm/h	89	46	25	110	18	20
C3, g/L	0.331	0.504	0.776	0.921	0.770	1.360
C4, g/L	<0.006	0.062	0.152	0.136	0.170	0.205
Anti-dsDNA, IU/mL	394.62	800	74.98	357.08	(-)	10.26
Anti-SS-A	(-)	(-)	(+++)	(+++)	(-)	(+++)
Anti-SS-B	(-)	(-)	(-)	(+++)	(-)	(++)
Anti-cardiolipin IgG, CU	(-)	(-)	(-)	28.3	(-)	(-)
Anti-β2-glycoprotein I IgG, CU	(-)	(-)	ND	118.6	(-)	(-)

No patient received IVIG or plasma exchange before LP. Systemic serological values represent measurements available at the index myelitis evaluation.

SLE-AM, systemic lupus erythematosus-associated myelitis; SLE-NMOSD overlap, systemic lupus erythematosus and neuromyelitis optica spectrum disorder overlap syndrome; anti-dsDNA, anti-double-stranded DNA; CSF, cerebrospinal fluid; CU, clinical units; ESR, erythrocyte sedimentation rate; WBC, white blood cell. Data notation: (+), positive; (-), negative. ND, not done.

Systemic serological findings also differed among individual patients. Patients 1 and 2 showed marked complement consumption accompanied by high anti-dsDNA antibody levels. In contrast, Patients 3–6 had more heterogeneous autoimmune serological profiles. Anti-SS-A antibodies were detected in Patients 3, 4, and 6, and anti-SS-B antibodies in Patients 4 and 6. Patient 4 additionally had positive antiphospholipid antibodies. Serum AQP4-IgG was positive in Patients 4–6 but was not tested in Patient 3. Other autoantibody findings are summarized at the individual-patient level in **Supplementary Table 1**.

Overall, higher routine CSF inflammatory indices were observed in Patients 1 and 2 at the available sampling time points, whereas Patients 3–6 showed greater serological heterogeneity, including AQP4-IgG positivity in Patients 4–6. Interpretation of the CSF differences is limited by heterogeneity in sampling timing and pre-LP immunotherapy exposure.

### 3.4 Treatment courses and clinical outcomes

Treatment strategies for each patient are summarized in **Table 3**, whereas longitudinal treatment courses and clinical outcomes are presented in **Figure 2**. Acute-phase treatment was mainly based on corticosteroids, frequently combined with cyclophosphamide (CTX) and/or intravenous immunoglobulin (IVIG). Patients 1–4 and 6 received intravenous methylprednisolone (IVMP), whereas Patient 5 received oral prednisone. CTX was administered during the acute phase in Patients 1–4 and 6, and IVIG was used in Patients 1, 3, 4, and 6. Maintenance treatment varied considerably among individual patients. Hydroxychloroquine was used in Patients 1–3, 5, and 6, and maintenance CTX was administered in Patients 1–4 and 6. Additional immunosuppressive agents included mycophenolate mofetil (MMF) in Patients 3 and 6, azathioprine in Patients 3 and 4, leflunomide in Patients 2 and 3, and tacrolimus in Patient 6. Several patients, particularly those with SLE-NMOSD overlap, required sequential or combination immunosuppressive treatment during their disease course.

**Table 3:** Acute-phase, maintenance-phase, and targeted biological therapies of individual patients.

Characteristics	Non-overlap SLE-AM		SLE-NMOSD overlap				
	Patient 1	Patient 2	Patient 3	Patient 4		Patient 5	Patient 6
Acute-phase therapies							
Acute corticosteroids	IVMP 1000 mg/d × 3d	IVMP 500 mg/d ×3d	IVMP 500 mg/d × 3d	IVMP 500 mg/d × 3d	Prednisone 25 mg/d		IVMP 500 mg/d × 3 d, 2 courses
IVIG	Yes (20 g/d ×5d)	No	Yes (20 g/d ×5d)	Yes	No		Yes
CTX (acute)	Yes	Yes	Yes	Yes	No		Yes
Maintenance-phase therapies							
HCQ	Yes	Yes	Yes	No	Yes		Yes
CTX (maintenance)	Yes	Yes	Yes	Yes	No		Yes
MMF	No	No	Yes	No	No		Yes
Azathioprine	No	No	Yes	Yes	No		No
Tacrolimus	No	No	No	No	No		Yes
Leflunomide	No	Yes	Yes	No	No		No
Targeted biological therapies							
Rituximab	Yes	No	No	No	No		No
Belimumab	No	No	No	No	No		Yes
Telitacicept	No	No	No	Yes	No		No
Inebilizumab	No	No	No	Yes	No		No

SLE-AM, systemic lupus erythematosus-associated myelitis; SLE-NMOSD overlap, systemic lupus erythematosus and neuromyelitis optica spectrum disorder overlap syndrome. CTX, cyclophosphamide; HCQ, hydroxychloroquine; IVIG, intravenous immunoglobulin; MMF, mycophenolate mofetil; IVMP, intravenous methylprednisolone.

**Figure 2:**
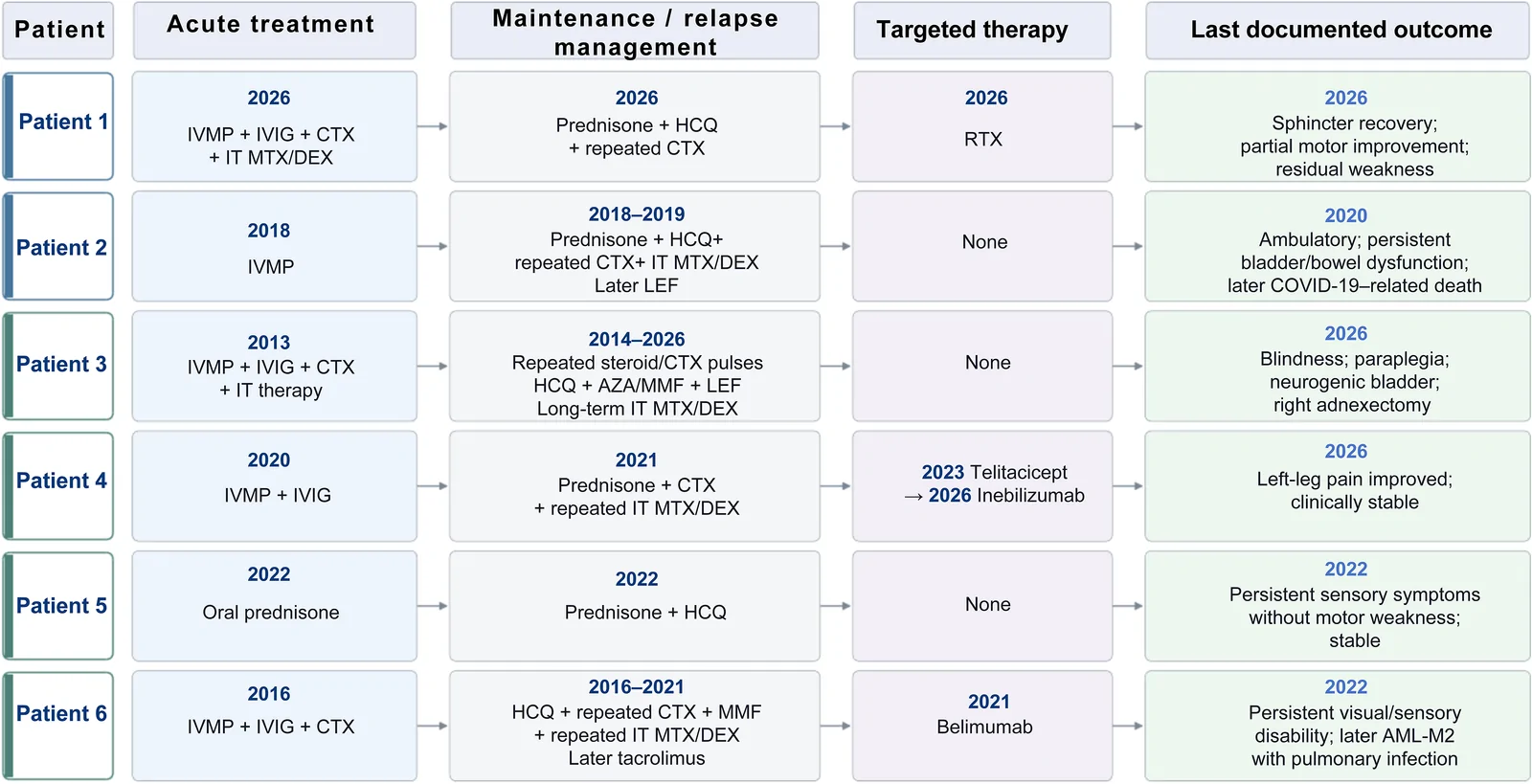
Treatment courses and clinical outcomes of the six patients. Timeline showing acute therapies, maintenance/relapse immunosuppressive regimens, targeted biological therapies, and the last documented clinical outcomes for all six patients. AZA, azathioprine; CTX, cyclophosphamide; DEX, dexamethasone; HCQ, hydroxychloroquine; IT, intrathecal; IVIG, intravenous immunoglobulin; IVMP, intravenous methylprednisolone; LEF, leflunomide; MMF, mycophenolate mofetil; MTX, methotrexate; RTX, rituximab. AML-M2, acute myeloid leukemia with maturation.

Targeted biological therapies were introduced in selected patients with persistent or recurrent disease manifestations. Patient 1 received rituximab, and Patient 6 received belimumab. Over a 5-year disease course, Patient 4 had experienced over 10 hospitalizations (averaging one flare every six months) characterized by recurrent limb numbness, weakness, and disabling pain, with pain being the major factor severely impairing her quality of life. Following the transition to inebilizumab, she achieved marked relief from pain and has remained completely relapse-free for 7 months. Her latest outpatient follow-up in September 2026 confirmed sustained clinical stability and no new disease activity, and she remained independently ambulatory. Given that this observation was based on a single patient, it should be interpreted as an individual clinical response rather than evidence of treatment efficacy.

Clinical outcomes were variable and often complicated by severe long-term morbidity. Patient 1 had severe residual neurological deficits. Patient 2 remained ambulatory with persistent sphincter dysfunction and later died from COVID-19-related complications. Patient 3 developed blindness, paraplegia, and neurogenic bladder, with ovarian cancer also documented during follow-up. Patient 5 had persistent sensory deficits without motor weakness, whereas Patient 6 had persistent visual and sensory disability and later developed acute myeloid leukemia with maturation (AML-M2) complicated by severe infection. Overall, the treatment courses and clinical outcomes varied substantially among the six patients, with several patients requiring prolonged, sequential, or targeted immunotherapy.

## 4 Discussion

### 4.1 Key findings and immunological implications

In this retrospective case series, we observed differing clinical, CSF inflammatory, and systemic immunological profiles among patients with non-overlap SLE-AM and SLE-NMOSD overlap syndrome. Patients 1 and 2 developed myelitis at SLE onset, had sphincter dysfunction, and showed higher routine CSF inflammatory indices at the available sampling time points, together with complement consumption and high anti-dsDNA antibody levels. By contrast, Patients 3–6 had more heterogeneous temporal courses; serum AQP4-IgG was positive in Patients 4–6, whereas the result was unavailable in Patient 3. Although Birnbaum et al. (4) originally highlighted gray-versus-white matter phenotypes in lupus myelitis, our observations suggest that serological and CSF findings may provide additional information for characterizing clinical heterogeneity. This interpretation is concordant with more recent biomarker-era reassessment of rheumatologic myelitis, which has emphasized that historical umbrella labels may group biologically different disorders (9).

### 4.2 Potential mechanistic hypotheses underlying the observed heterogeneity

Our observations support the clinical value of considering NMOSD-related features and serum biomarkers when evaluating myelitis in patients with SLE. Although prior meta-analyses have reported inconsistent prognostic value for antiphospholipid antibodies, complement levels, and other conventional markers, these discrepancies may partly reflect the mixing of biologically different myelitis phenotypes within prior cohorts (13). Based on these data-supported findings, we now turn to potential mechanistic hypotheses. The combination of marked CSF pleocytosis, elevated protein, high anti-dsDNA titers, and profound C3/C4 depletion in Patients 1 and 2 leads us to hypothesize a potential link to immune-complex-driven inflammatory injury and complement activation (13–15). One possible literature-informed hypothesis is that immune-complex deposition along the spinal microvasculature may contribute to vascular inflammation and subsequent blood-spinal cord barrier disruption (2, 15). In line with this, endothelial activation, complement deposition, Fc receptor-mediated inflammation, and cytokine-driven barrier disruption are well-described mechanisms of neuroimmune injury at the CNS interface in SLE (14, 15). Accordingly, the marked CSF inflammatory pattern observed in Patients 1 and 2 may reflect the interaction between systemic immune activation and localized spinal cord inflammation through blood-spinal cord barrier disruption.

Based on the data-supported finding of serum AQP4-IgG positivity in Patients 4–6, we hypothesize that NMOSD-related astrocyte-targeted immune-mediated injury may represent an underlying mechanism in this subset of SLE-NMOSD overlap patients. While AQP4-IgG-mediated astrocytic injury, complement-dependent cytotoxicity, and secondary demyelination are well-established central features of NMOSD pathobiology, which are often accompanied by modestly abnormal routine CSF cell counts (16, 17), the precise *in vivo* dynamics in our patients remain unmeasured. Specifically, we postulate as a mechanistic hypothesis that a chronic systemic autoimmune state in AQP4-IgG-positive patients may facilitate periodic access of circulating AQP4-IgG to the CNS, after which locally restricted complement-mediated injury can occur at astrocytic foot processes along the spinal microvasculature (15–17). Although this pathogenetic framework is inferred from serological patterns rather than direct histopathological demonstration, it offers a conceptual model to help explain the clinical dissociation between relatively modest routine CSF inflammatory indices and substantial neurological disability. Consequently, in AQP4-IgG-positive cases, established NMOSD pathobiology provides a plausible framework for considering astrocyte-targeted injury as one potential contributor to neurological damage, supporting further evaluation of mechanism-informed therapeutic strategies.

### 4.3 Shifting toward mechanism-tailored therapeutic strategies

In current practice, SLE-AM is primarily managed with glucocorticoids, cyclophosphamide, and other broad immunosuppressive agents (2, 13, 18). In our case series, substantial long-term neurological morbidity persisted in several patients despite intensive immunosuppressive therapy, highlighting the importance of timely treatment and the limitations of empiric, non-stratified approaches. Regarding therapeutic strategies, Patients 1 and 2 with non-overlap SLE-AM, who showed marked systemic lupus activity and strongly inflammatory CSF findings, appeared to fit an acute inflammatory-humoral model in which rapid suppression of systemic immune activation remains biologically reasonable (2, 13, 17). In contrast, for AQP4-IgG-positive Patients 4–6, the underlying NMOSD pathobiology supports consideration of mechanism-tailored immunotherapies rather than reliance solely on conventional NPSLE treatment strategies. Following a switch to inebilizumab, an anti-CD19 monoclonal antibody (17, 19), Patient 4 reported further relief of disabling pain and experienced a period without documented neurological relapse through September 2026, representing a clinically relevant period of stability compared with her previous recurrent course. Recent case-based experience has also described inebilizumab use in patients with coexisting SLE and NMOSD (20). Together, these observations suggest that CD19-directed B-lineage therapy may have potential value in selected patients with SLE-NMOSD overlap and support further prospective evaluation.

Targeted biological therapies represent an evolving area of interest, although their application in this setting remains variable. B-cell depletion or broader B-lineage targeting may be relevant in overlap disease, as AQP4-IgG-associated pathology is intrinsically linked to pathogenic humoral immunity and plasmablast survival pathways (16, 17, 19). At present, the use of BAFF/APRIL-directed therapies, complement inhibitors, and IL-6 blockade in refractory SLE-associated NMOSD is largely based on anecdotal cases or limited cohort-level data (16, 17, 21). Similarly, while belimumab is effective in systemic SLE, its benefit in preventing neuropsychiatric flares has not been consistently demonstrated (22). The variability in therapeutic responses reported in previous studies may partly reflect clinical and biological heterogeneity among patients with SLE-associated myelitis. Accordingly, future studies should evaluate whether biomarker-informed therapeutic selection can improve outcomes, particularly in patients with confirmed AQP4-IgG-positive NMOSD overlap.

### 4.4 Limitations and future directions

Several limitations of this study warrant consideration. First, the small sample size and retrospective single-center design limit generalizability and preclude formal comparative inference. Diagnostic classification also relied on the clinical, serological, CSF, and imaging data available in the medical records, with some biomarker data unavailable in individual patients. Second, CSF sampling timing and pre-LP immunotherapy differed across patients and may have influenced routine inflammatory indices, limiting attribution of between-patient differences solely to disease phenotype. Standardized systemic and neurological outcome measures, including SLEDAI-2K and EDSS, were also not routinely recorded, so longitudinal disease activity and functional outcomes were described using the available clinical and laboratory data. Third, proposed mechanisms such as immune-complex/complement-mediated vascular injury, blood-spinal cord barrier dysfunction, and astrocytic injury were not directly measured and should be regarded as literature-informed hypotheses. Finally, treatment regimens and follow-up duration varied, limiting causal interpretation of individual treatment responses, particularly the post-inebilizumab course of Patient 4. Future prospective multicenter studies with standardized sampling, imaging, biomarkers, and longitudinal outcome assessment are warranted.

## 5 Conclusion

Our case series illustrates clinical and immunological heterogeneity among patients with SLE-associated myelitis. Patient-level assessment of CSF inflammatory findings, complement activity, AQP4-IgG status, clinical course, and MRI features may help distinguish different patterns of myelitis in patients with SLE. These observations support further investigation of biomarker-informed, individualized treatment strategies in larger prospective studies.

## Data Availability

The raw data supporting the conclusions of this article will be made available by the authors, without undue reservation.
