# Supplementary material for: Phenotypic and immunological profile divergence between SLE-associated myelitis and SLE-NMOSD overlap syndrome: a retrospective case series
Source: Front Immunol. 2026 Oct 20;17:1925485. doi: 10.3389/fimmu.2026.1925485 (PMC13727460; doi:10.3389/fimmu.2026.1925485)
Supplement: Supplementary file 1 [file Table1.docx]

**Supplementary Table 1. Other serological markers of individual patients**

| **Characteristics** | **Non-overlap SLE-AM** | | **SLE-NMOSD Overlap** | | | |
| --- | --- | --- | --- | --- | --- | --- |
|  | **Patient 1** | **Patient 2** | **Patient 3** | **Patient 4** | **Patient 5** | **Patient 6** |
| ANA titer | 1:3200 | 1:1000 | 1:1000 | 1:3200; | 1:320 | 1:1000 |
| Anti-Sm | >500 | ++ | + | + | (-) | (-) |
| Anti-nRNP/Sm | >500 | +++ | (-) | + | (-) | (-) |
| Anti-histone | 77.9 U/mL | ++ | (-) | ++ | (-) | (-) |
| Anti-nucleosome | 301.0 U/mL | + | (-) | +++ | ND | (-) |
| Anti-ribosomal P | >500 | (-) | (-) | (-) | + | (-) |
| Anti-Ro-52 | >500 | (-) | +++ | +++ | (-) | +++ |
| Lupus anticoagulant | (-) | (-) | (-) | (+) | (-) | (-) |

**Abbreviations:** ND, not done; **Data notation:** (+), positive; (-), negative.
